# Lipid Anti-Lipid Antibody Responses Correlate with Disease Activity in Systemic Lupus Erythematosus

**DOI:** 10.1371/journal.pone.0055639

**Published:** 2013-02-07

**Authors:** Vojislav Jovanović, Nurhuda Abdul Aziz, Yan Ting Lim, Amanda Ng Ai Poh, Sherlynn Jin Hui Chan, Eliza Ho Xin Pei, Fei Chuin Lew, Guanghou Shui, Andrew M. Jenner, Li Bowen, Eoin F. McKinney, Paul A. Lyons, Michael D. Kemeny, Kenneth G. C. Smith, Markus R. Wenk, Paul A. MacAry

**Affiliations:** 1 Immunology Programme and Department of Microbiology, National University of Singapore, Singapore; 2 Cambridge Institute for Medical Research, Cambridge, United Kingdom; 3 Department of Medicine, University of Cambridge, School of Clinical Medicine, Addenbrooke’s Hospital, Cambridge, United Kingdom; 4 Department of Biochemistry, National University of Singapore, Singapore; 5 School of Biological Sciences, Illawara Health and Medical Research Institute, University of Wollongong, Australia; MUSC SC College of Pharmacy, United States of America

## Abstract

Systemic Lupus Erythematosus (SLE) is a chronic autoimmune disorder characterized by broad clinical manifestations including cardiovascular and renal complications with periodic disease flares and significant morbidity and mortality. One of the main contributing factors to the pathology of SLE is the accumulation and impaired clearance of immune complexes of which the principle components are host auto-antigens and antibodies. The contribution of host lipids to the formation of these autoimmune complexes remains poorly defined. The aim of the present study was to identify and analyze candidate lipid autoantigens and their corresponding anti–lipid antibody responses in a well-defined SLE patient cohort using a combination of immunological and biophysical techniques. Disease monitoring in the SLE cohort was undertaken with serial British Isles Lupus Assessment Group (BILAG) scoring. Correlations between specific lipid/anti-lipid responses were investigated as disease activity developed from active flares to quiescent during a follow up period. We report a significant negative correlation between anti-lipid antibodies for 24S-hydroxycholesterol, cardiolipin and phosphatidylserine with SLE disease activity. Taken together, these data suggest that lipid autoantigens represent a new family of biomarkers that can be employed to monitor disease activity plus the efficacy of therapeutic intervention in SLE.

## Introduction

Systemic Lupus Erythematosus (SLE) is a chronic inflammatory autoimmune disease found predominantly in women. Complex interactions amongst immune, genetic, environmental and hormonal factors have been implicated in SLE susceptibility and pathogenesis [1]. Numerous mouse and human studies have implicated dysfunctional cellular and immune components including autoimmune T and B lymphocytes [2], [3], [4]; elevated levels of pro- inflammatory cytokines [5]; formation of antinuclear antibodies [6]; accumulation and impaired clearance of post-apoptotic cell remnants [7], [8] or failure of FcγR-mediated clearance of immune complexes [9] in the pathology of Systemic Lupus Erythematosus.

The role of lipids and anti-lipid responses in Systemic Lupus Erythematosus and other autoimmune diseases remains poorly defined in comparison to proteins and genetic factors based on the technical challenges inherent in their analysis. A summary of studies linking oxysterols, phospholipids and prostaglandin derivatives with autoimmune, degenerative and age-related diseases including SLE is provided in Table 1. Thus there is a requirement for a broader and more detailed analysis of the role of lipids in these diseases.

**Table 1:** A summary of reported lipids and anti-lipid antibodies involved in autoimmune, degenerative and age-related diseases.

Pathologies	Organ involved	Associated oxidizedlipid/anti-lipid Ab	Origin of oxidizedlipid	Method of detection	Reference
Alzheimer disease, vascular demented patients	CNS	24S-hydroxycholesterol	Plasma	ID-MS	[14]
Alzheimer disease	CNS	24S-hydroxycholesterol	Plasma, cerebrospinalfluid	ID-MS	[49]
Multiple sclerosis	CNS	24S-hydroxycholesterol	Plasma, cerebrospinalfluid	ID-MS	[15]
Alzheimer disease	CNS	27-hydroxycholesterol	Brain tissue	GC-MS	[50]
Hereditary spastic paresis	CNS	27-hydroxycholesterol, 25-hydroxycholesterol	Plasma, cerebrospinalfluid	ID-MS	[51]
Atherosclerosis	Cardiovascular	27-hydroxycholesterol, 25-hydroxycholesterol, 7β- hydroxycholesterol	Plasma	HP-LC	[52]
Parkinson disease	CNS	F(2)-isoprostanes, hydroxyeicosatetraenoicacid products, 7β- hydroxycholesterol, 27-hydroxycholesterol, 7-ketocholesterol, neuroprostanes(F(4)NPs)	Plasma	GC-MS	[17]
Multiple sclerosis	CNS	7-ketocholesterol	Serum, CSF	ID-MS	[53]
Multiple sclerosis	CNS	Oxidized phosphatidylcholine (OxPC)Anti-OxPC (T15 Idiotype) antibodies	Brain extracts, CSF	Western blotting,	[16]
SLE	Different organ systems	Anti-cardiolipin Ab	Serum	ELISA	[54]
Immunoglobulin A deficiency	Different organ systems	Anti-cardiolipin Ab	Serum	ELISA	[55]
Antiphospholipid syndrome	Different organ systems	Anti-cardiolipin Ab	Serum	ELISA	[56]
Systemic Lupus Erythematosus	Different organ systems	15-F2t-IsoP	Serum	ELISA	[57]
Systemic Lupus Erythematosus	Different organ systems	15-F2t-IsoP	Plasma	GC-MS	[58]
Systemic Lupus Erythematosus; Antiphospholipid syndrome	Cardiovascular system	Anti-phosphatidylserine Ab,Anti-cardiolipin Ab	Plasma	ELISA	[59]
Alzheimer disease	CNS	24S-hydroxycholesterol	Plasma	LC-MS	[60]

Oxysterols represent the family of host lipids most strongly implicated in autoimmune conditions (Table 1). These are oxygenated derivatives of cholesterol that are intermediates in the cholesterol excretion pathway [10]. Cholesterol oxidation is either through attack by reactive oxygen species (ROS) that oxygenate the sterol ring at the C7-position or by enzymatic hydroxylation of cholesterol side-chains that generate 24S-, 25- and 27-hydroxycholesterol respectively [11]. 24S-hydroxycholesterol is specifically generated in the central nervous system [12]–[13] and plasma levels of this lipid have been implicated in diseases linked to CNS inflammation including Alzheimer’s and Vascular dementia [14].

Elevated plasma levels of 24S-hydroxycholesterol was reported in Multiple Sclerosis (MS) patients with positive cranial MRI scans indicating an acute inflammatory episode of demyelination [15]. Oxidized phosphatidylcholine and their corresponding autoantibodies have also been implicated in MS [16] **.** Other lipid markers including F(2)-isoprostanes, 7-β-hydroxycholesterol, 7-ketocholesterol and 27-hydroxycholesterol have been linked to Parkinson’s disease [17]. 7-ketocholesterol may also be involved in the pathophysiology of atherosclerosis where it is suspected of inducing apoptosis in the cells of the vascular wall including monocytes/macrophages [18]. This lipid is also known to be related to oxidized-LDL-mediated cytotoxicity [19]. 7-β hydroxycholesterol is proposed to promote human NK cell death and may also be involved in atherosclerosis [20]. This study focuses on the role of oxidized lipids and anti-lipid responses in Systemic Lupus Erythematosus (SLE).

## Materials and Methods

### Patients

The patient cohort employed was composed of individuals referred to Addenbrooke’s Hospital, Cambridge, UK between 2004 and 2008. All patients provided written informed consent and ethical approval was obtained from the Cambridge Local Research Ethics Committee (Ref: 04/023). Blood was collected at two time points: the moment of disease [21] - flare; and during the follow-up period. Follow-up was defined as the period between 3-months and 12-months post therapy. Disease monitoring was undertaken with serial BILAG scoring [22]. All patients were enrolled with active disease with an average BILAG score of 16.01 prior to treatment. Patients on treatment entered clinical remission and the average BILAG score in the follow up period was reduced to 2.4+/−2.1. For all patients, a full haematological, biochemical and immunological profiling was done [21]. BILAG scores for 3 time-points and clinical data for patients with flare are included (Supplementary table). Thirteen paired SLE patients’ samples were used to analyze changes in lipid and anti-lipid IgG levels between the flare and follow-up period (between 3–12 months post-therapy). Twenty patients with flare (including 13 previously mentioned patients) were used for the correlation analysis.

### Blood Processing

Blood was collected in EDTA tubes and peripheral blood mononuclear cells (PBMC) separated on Ficoll-Paque PLUS gradient (GE Healthcare, Sweden). Plasma was stored at −80°C prior to use.

### Lipid Standards and Chemicals

Phosphatidylcholine, oxidized phosphatidylcholine, cardiolipin, phosphatidylserine, were obtained from Avanti Polar Lipids (Alabaster, AL, USA). 7-ketocholesterol, 7 alpha-hydroxycholesterol, 7 beta-hydroxycholesterol, and cholesterol which were purchased from Sigma (St. Louis, MO, USA), 24S-hydroxycholesterol was obtained from Steraloids (Newport, RI, USA). 7-α-hydroxycholesterol-d7, 7-β-hydroxycholesterol-d7, 26 (27)-OH cholesterol-d5, 24S-hydroxycholesterol-d6, 7-ketocholesterol-d7, lathosterol, and lathosterol-d4 were purchased from CDN Isotopes (Quebec, Canada). Arachidonic acid, arachidonic acid-*d_8_*, 8-Iso-PGF_2_a (8-*iso*-prostaglandin F_2a_ or 15-F_2t_-IsoP or iPF_2a_-III), 8-iso-PGF_2a_-*d_4_*, 8-F_2t_-IsoP-d_4_ (iPF2_a_-IV or 5*S*,9a,11a,-trihydroxy-1a,1b,1g-trihomo-18,19,20-trinor-8b-prosta-2Z,6E-dien-1-oic acid) and 5-F_2t_-IsoP (iPF_2a_-VI-*d_4_* or 5*S*,9a,11a,-trihydroxy-(8b)-prosta-6E,14Z-dien-1-oic acid) were purchased from Cayman Chemical (Ann Arbor, MI USA). All solvents used were HPLC grade.

### Enzyme-linked Immunosorbent Assay (ELISA) and Lipid Quantification

Maxi-sorp plates (NUNC, Denmark) were coated with 5 ug/ml of lipids in EtOH evaporated for 2 hrs at RT. Plates were blocked with 0.8% collagen in PBS (Sigma-Aldrich, USA). Anti-lipid IgG responses were detected using goat-anti human IgG antibody conjugated with horseradish peroxidase (HRP) (Thermo Scientific Pierce, USA). TMB substrate (BD OptEIA™, BD Biosciences) was employed for 15 mins and colour development assayed at 450 nm using a Perkin-Elmer Victor^3^V plate reader.

### Lipid Extraction and Gas Chromatography-mass Spectrometry (GC- MS)

Lipids were extracted using a modified Folch method and hydrolysed in methanolic KOH [23]. COPs and F_2_-Isoprostanes were then purified into 2 separate fractions by solid phase extraction on mixed mode anion exchange columns, dried under a stream of nitrogen, derivatised and injected onto an Agilent 5973/6890 GC-MS system as described previously [24]. Briefly, cholesterol oxidative products (COPs) were derivatised to generate their trimethyl silyl ether derivatives and analysed in the EI (electron ionization) mode. F_2_-isoprostanes were derivatised to form their pentafluorobenzyl (PFB) ester, trimethyl silyl ether derivatives and analysed in the NCI (negative chemical ionization) mode. Quantification was achieved by relating peak area of samples with respective deuterated internal standards according to the previously published method [25]. 8-Iso-PGF_2_a and 5-F_2t_-IsoP F_2_-isoprostane isomers co-eluted and were measured together as a single peak.

### Lipid Extraction and Liquid Chromatography – Mass Spectrometry (LC – MS)

Phospholipids were extracted according to a modified protocol of Bligh and Dyer, 1959 [26]. Synthetic lipids obtained from Avanti Polar Lipids (Alabaster, AL, USA) were spiked as internal standards. The extracted lipids were measured using ABI 3200QT (Applied Biosystems, Foster City, CA) interfaced to a HPLC system using multiple reaction-monitoring mode [27]. Phospholipids were separated on a Phenomenex Luna 3 µ C18 column (150 mm×2 mm)(Phenomenex, Torrance, CA, USA). Signal intensities obtained for each lipid class were normalized to the appropriate internal standard.

### Statistical Analysis

Differential levels of lipids or anti-lipid IgGs between time points were assessed in Prism (GraphPad Software) using the Wilcoxon nonparametric matched pairs test with a p<0.05 value considered significant in all cases. A correlation between different clinical parameters and anti-lipid IgG levels was examined using a Spearman nonparametric correlation and Linear regression tests.

## Results

### Total IgG Levels in SLE Patients Remain Unchanged during Therapy

**Figure 1:**
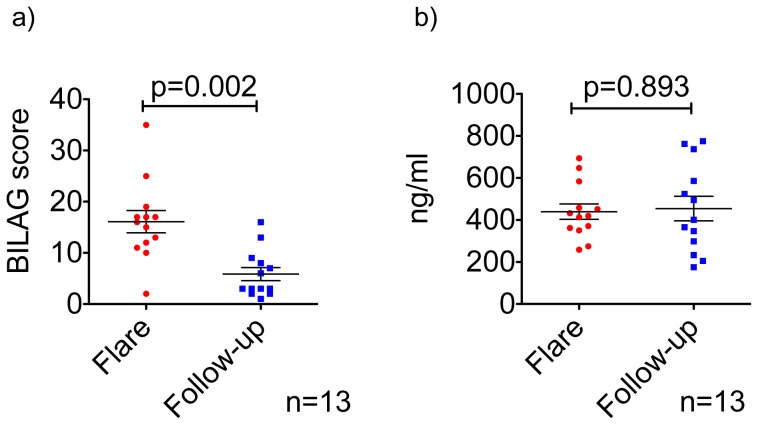
SLE disease activity score measured with the BILAG index reduces significantly over the time while total IgG levels remain the same. The British Isles Lupus Assessment Group (BILAG) index score during disease flare and the follow-up period. Figure 1a. A significant improvement is detectable over the time. Figure 1b. A level of total IgGs in patients’ blood is similar during disease flares and in the follow-up period. p<0.05 value considered significant in Wilcoxon test.

A reduction in BILAG score was employed as the principal indicator for treatment success in our SLE patient cohort. We observed a decrease in this score as patients progressed from flare through follow-up period (Fig. 1a). Absolute levels of IgG in the patients’ plasma remain unchanged at these time points (Fig. 1b).

In Table S1, we present BILAG scores for 8 systems: General (Gen), Mucocutaneous (Muc), Neurological (Cns), Musculoskeletal (Msk), Cardiovascular and Respiratory (Car), Vasculitis (Vas), Renal (Ren) and Haematological (Hae). As documented in this table, BILAG scores in SLE patients at 3-months and 12-months indicate that these patients have quiescent disease and can thus be combined as our follow-up cohort.

### SLE Patients with Active Disease Exhibit Increased Levels of Lipid and Anti-lipid Responses

**Figure 2:**
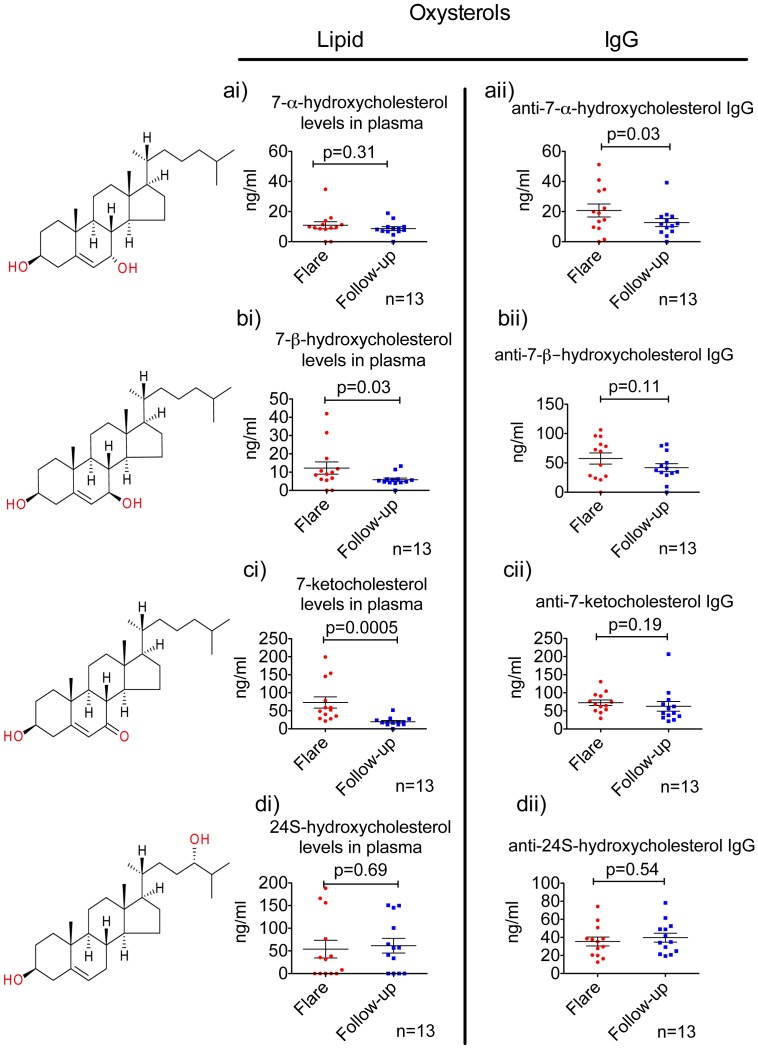
Levels of oxysterol and anti-oxysterol IgG in SLE patients’ plasma. Analysis by GC*-*MS and ELISA show higher levels of oxidized cholesterols or anti-cholesterol IgGs in patients during flare versus follow-up period. Figure 2ai and 2 aii. 7-α-hydroxycholesterol levels and anti-7-α-hydroxycholesterol IgG levels in plasma. Figure 2bi and 2bii. 7-β-hydroxycholesterol levels and anti-7-β-hydroxycholesterol IgG levels in plasma. Figure 2ci and 2cii. 7-ketocholesterol levels and anti-7-ketocholesterol IgG levels in plasma. Figure 2di and 2dii. 24S-hydroxycholesterol levels and anti-24S-hydroxycholesterol IgG levels in plasma. p<0.05 value was considered as significant in Wilcoxon test.

Gas chromatography-mass spectrometry (GC*-*MS) analyses indicate significantly higher levels of oxidized cholesterols in patients during flare versus follow-up. Specifically, 7-β-hydroxycholesterol, 7-ketocholesterol (Fig. 2bi, 2ci) were significantly increased. At the same time, 24S-hydroxycholesterol and 7-α-hydroxycholesterol levels remained unchanged (Fig. 2ai, 2di). Only five of the patients had a form of therapy aimed at reducing cholesterol levels-a combination of fenofibrate, pravastatin or simvastatin (Table S2). In four out of five patients, administration of lipid-lowering drugs started from the moment of flare and lasted over next 12 months. Administration of statins will reduce cholesterol levels. However, we have not addressed to what extent statins will affect levels of oxidized cholesterols over time in these patients. Anti-7-α-hydroxycholesterol IgG responses against oxysterols were significantly higher in patients with flare in comparison with the results obtained during the follow-up period (Fig. 2aii). The same trend was seen in anti-7-β-hydroxycholesterol and anti-7-ketocholesterol IgGs though these differences were not significant (Fig. 2bii and 2cii). Anti-24S-hydroxycholesterol IgG levels did not change over time (Fig. 2dii).

### Levels of Oxidatively Modified Unsaturated Fatty Acids and Anti-lipid Antibodies Change during Therapy

**Figure 3:**
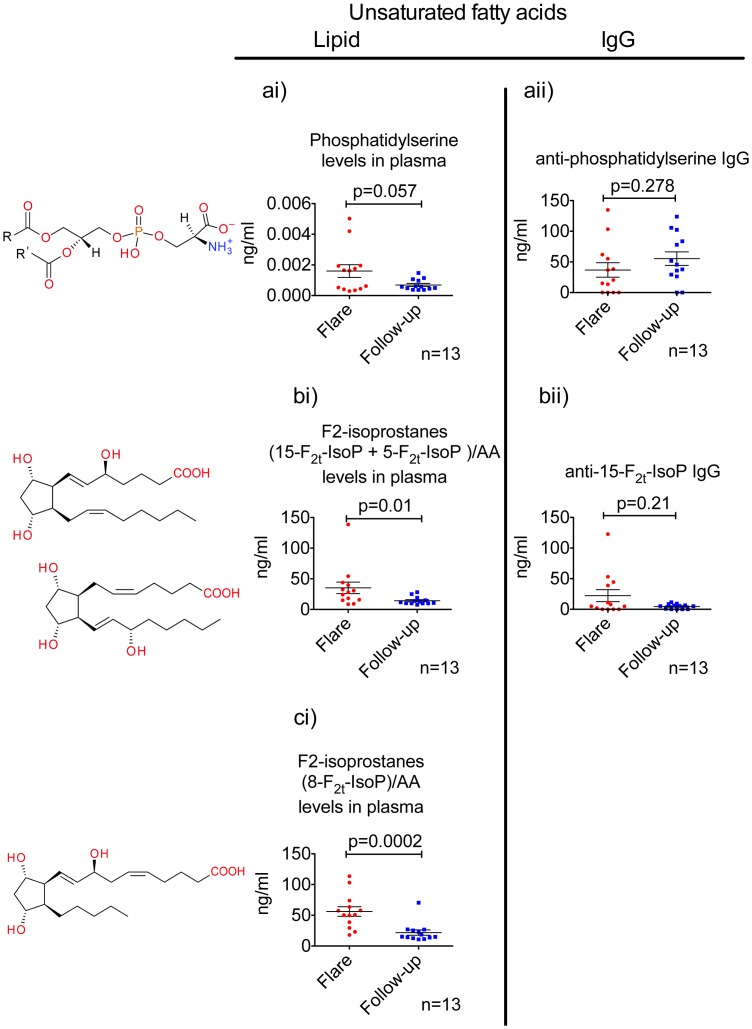
Levels of unsaturated fatty acids and anti-unsaturated fatty acids IgG in SLE patients’ plasma. Figure 3ai and 3aii. Phosphatidylserine levels and anti-phosphatidylserine IgG levels in plasma. Figure 3bi and 3bii. Isoprostane (15-F_2t_-IsoP +5-F_2t_-IsoP) levels normalized against AA values and anti-15-F_2t_-IsoP IgG levels in plasma. Figure 3ci. Isoprostane (8-F_2t_-IsoP) levels normalized against AA values. p<0.05 value was considered as significant in Wilcoxon test.

Mass-spectroscopy analyses of phosphatidylserine during the flare and follow-up period suggest a reduction in this lipid over time (Fig. 3ai). Also, anti-phosphatidylserine IgG levels tend to increase during the follow-up period (Fig. 3aii).

Normalized values for isoprostanes (normalized against arachidonic acid (AA)) significantly decrease from the moment of flare (Fig. 3bi and 3ci). We employed 15-F2t-IsoP as the antigen in this assay where we measured anti-isoprostane IgG response (Fig. 3bii). Anti-15-F2t-IsoP IgG exhibited a trend of higher levels in flare compared to follow-up.

### Anti-lipid IgG Levels for Other Lipids also Change during Therapy

**Figure 4:**
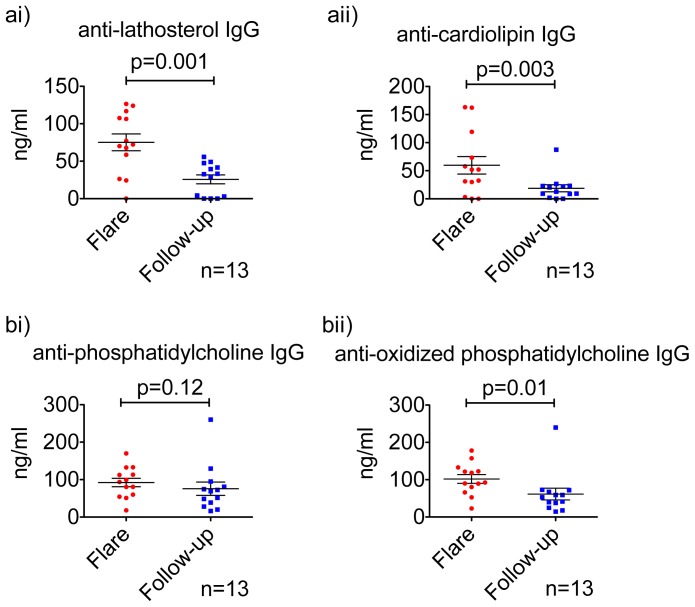
Anti-lipid responses against lathosterol, cardiolipin, phosphatidylcholine and oxidized-phosphatidylcholine. Figure 4ai anti-lathosterol IgG levels in plasma. Figure 4aii anti-cardiolipin IgG levels in plasma. Figure 4bi anti-phosphatidylcholine IgG levels in plasma. Figure 4bi anti-oxidized phosphatidylcholine IgG levels in plasma. p<0.05 value considered significant in Wilcoxon test.

Lathosterol is a precursor of cholesterol and we detected significantly higher anti-lathosterol IgG responses in patients with flare (Fig. 4ai). Phospholipids and anti-phospholipid antibodies are often seen in autoimmune disorders and are associated with cerebrovascular disease or moderate-to-severe cognitive dysfunction. In our study we detected significantly higher levels of anti-cardiolipin (Fig. 4aii) and anti-oxidized phopsphatidylcholine in flare (Fig. 4bii). Anti-phopsphatidylcholine IgG in patients with flare was also increased (Fig. 4bi).

### Levels of Anti-lipid Antibodies Negatively Correlate with BILAG Scores

**Figure 5:**
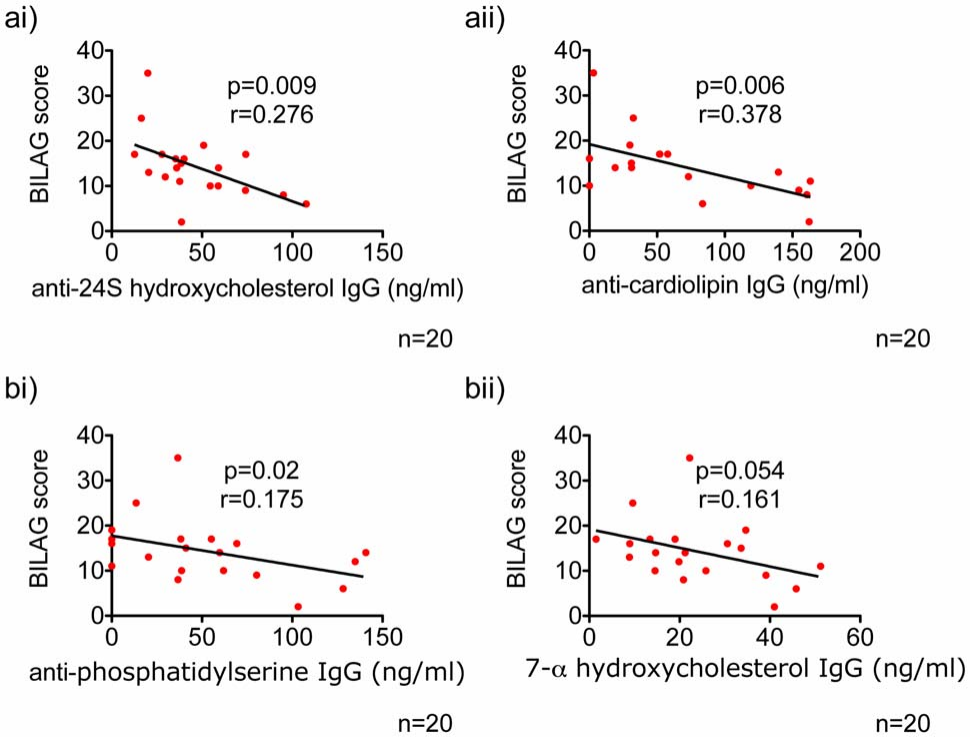
Biomarker candidates that correlate with BILAG scores. Figure 5ai, 5bi, 5ci. Anti-24S hydroxycholesterol IgG, anti-cardiolipin IgG and anti-phosphatidylserine IgG levels negatively correlate with BILAG scores. Figure 5bii. Anti-7-α-hydroxycholesterol IgG levels also show trend of negative correlation with BILAG scores. p<0.05 value was considered as significant in Spearman correlation and Linear regression tests.

We analyzed our lipid anti-lipid data for correlations with a score of disease activity in SLE. For those analyses we included an additional 7 SLE patients for which we had BILAG scores during the flare. In total we analyzed 20 patients. Spearman correlation and linear regression tests showed that anti-24S hydroxycholesterol IgG, anti-cardiolipin IgG and anti-phosphatidylserine IgG, negatively correlate with BILAG score – the disease activity index (Fig. 5ai, 5aii, 5bi respectively). A similar, although not significant trend was seen in anti-7-α hydroxycholesterol IgGs (Fig. 5bii). A higher level of these anti-lipid antibodies was observed in patients with lower BILAG scores suggesting that these may be markers for improved prognosis.

## Discussion

In SLE, the heterogeneity of disease presentations in patient populations combined with a poor understanding of the underlying pathological mechanisms result in challenges for successful disease management. The American College of Rheumatology classification utilizes 11 criteria for diagnosis of lupus, of which a patient must meet four [28]. Several computerised indices for measuring clinical disease activity in Systemic Lupus Erythematosus are employed in clinical practice- BILAG, SLEDAI, LUMINA [29]–[32]. Despite advances in treatment protocols there remains a dearth of good diagnostic and prognostic biomarkers to facilitate improvements in disease characterization and management [33]–[35]. Moreover, the immune modulating treatment used for SLE remains a significant problem with many patients developing treatment-associated complications [36]. Thus, the identification of biomarkers that correlate with a good response to therapy will have an impact on treatment-associated side-effects where less-aggressive approaches can be employed [33].

In our lipidomic and immunologic approaches we have identified a new corpus of biomarkers relevant to this disease. This study focused on a clinically well-defined SLE cohort that was monitored over a period of 12 months. Based on clinical score and statistical tests, we grouped samples provided between 3 and 12 months post-therapy as our follow-up cohort. This approach enabled us to apply statistical tests for paired samples.

We were also interested in correlations between lipid/anti-lipid IgG levels and BILAG scores during disease flares. In this case we did not need paired samples and thus employed 20 samples in total.

We have identified new targets that correlate with disease score. Our approach employed gas chromatography-mass spectrometry (GC*-*MS) and liquid chromatography-mass spectrometry (LC*-*MS) to investigate the lipids of interest. The presence of anti-lipid autoantibodies was confirmed by an enzyme*-*linked immunosorbent assay where we focused on the immunoglobulin isotype G.

Our high-resolution analysis has identified a cohort of new anti-lipid autoantibodies. Whilst total IgG levels remain consistent during therapy, we detected differential levels of anti-lipid IgGs for four different lipid species: lathosterol; enzymatically produced oxysterols 7-α-hydroxycholesterol and oxidatively modified phospholipids – cardiolipin, and oxidized phosphatidylcholine.

The measurement of oxygenated cholesterol species remains technically challenging based on the proposed auto-oxidation that can occur during lipid derivation, processing or storage from biological samples. [37]–[39]. In our study plasma levels of oxysterols showed a general trend of decrease over time. Thus, if auto-oxidation occurs, it should happen at a similar level in all samples tested without a significant influence on absolute ratios. The main conclusion of this study concerns relationships between anti-lipid antibodies and BILAG score. Anti-lipid antibodies identified in lupus patients’ plasma are part of an autoimmune response that targets lipid antigens. These develop as a result of pathogenic processes in the afflicted patients. Thus, antibodies from SLE patient’s plasma should not target oxidized cholesterols that are result of *in vitro* auto-oxidation processes (e.g. during the ELISA plate coating with lipid-antigen). We found similar levels of anti-24S-hydroxycholesterol IgGs between flare and follow-up (Fig. 2dii). There was no significant difference in IgG levels against 27-hydroxycholesterol when analyzing the same time points (data not shown). Phosphatidylserine levels in plasma showed a trend of reduction over the time. Anti-cardiolipin antibodies are one of several anti-phospholipid antibodies that have been previously identified in SLE patients [40] where cardiolipin present on the surface of apoptotic cells acts as an immunologic trigger for the production of the autoantibodies [41].

Isoprostanes are generated by the free radical-mediated peroxidation of arachidonic acid (AA) [42]. 15-F2t-IsoP is a marker of free radical damage and lipid peroxidation *in vivo* that is formed by free radical catalysis of arachidonic acid [43]. Serum levels of 15-iso-PGF2alpha and 8-iso-PGF2alpha in SLE patients showed a significantly higher level at flare compared to the post-therapy period.

BILAG is currently accepted as the best disease activity score in SLE [22] and thus we analyzed which if any of our lipid/anti-lipid parameters correlate. We observed that anti-phosphatidylserine, anti-cardiolipin and anti-24S hydroxycholesterol IgG negatively correlate with the BILAG score. Anti-7-α-hydroxycholesterol IgGs also show trend of negative correlation with BILAG score. However, for this anti-lipid response we were not able to confirm a statistically significant correlation.

At the same time points we were not able to detect statistically significant correlations between BILAG scores and one traditional SLE biomarker - anti-DNA antibodies (data not shown). This can potentially be explained by the fact that anti-DNA antibodies are found in only 60% of SLE patients and those antibodies are particularly associated with lupus nephritis [44].

The negative correlations observed can be explained by two possible mechanisms: the presence of anti-lipid IgG during flare may be beneficial and aid immune complex clearance through IgG receptors expressed on phagocytic cells such as the Kuppfer cells in the liver [45]–[46]; or reduced levels of lipids and their corresponding anti-lipid antibodies in plasma are a consequence of deposition of immune complexes in the tissues. Both of these mechanisms are currently under investigation in our laboratory.

It is an interesting observation that levels of anti-lipid IgGs for 24S-hydroxycholesterol and phosphatidylserine remained very similar over the time period of study. Since levels of these antibodies negatively correlate with BILAG score at the time of flare, one explanation could be that their presence during the post-flare period might have a protective role. For the actual levels of phosphatidylserine we saw a trend of reduction between flare and follow-up. This phenomenon suggests that either oxidation processes in unsaturated fatty acids were reduced or the development of phosphatidylserine immune complexes intensified over time.

IgM autoantibodies have been linked to apoptosis-associated antigen clearance [47]–[48]. IgM anti-phosphorylcholine was shown to be notably higher in patients with low SELENA-SLEDAI disease activity index [47]. Those patients encountered less cardiovascular and renal problems and high levels of IgM are attributed to the homeostatic and protective roles [47]. The panel of auto-antigens that appear during the apoptosis is large and further identification of protective IgG and IgM antibodies is required.

Clinical and laboratory markers currently used in SLE have moderate utility based on specificity and sensitivity. Based on our findings, we suggest that the measurement of anti-lipid IgGs for 24S-hydroxycholesterol, cardiolipin and phosphatidylserine may be used as a sensitive and non-invasive method of surveillance during treatment. These therefore represent improved biomarkers for the evaluation and development of better therapeutic strategies aimed at reducing treatment associated morbidity and mortality, a significant problem in SLE.

## Supporting Information

Table S1 **BILAG scores summary for three different time-points.** BILAG scores (A-E) for 8 headings: General (Gen), Mucocutaneous (Muc), Neurological (Cns), Musculoskeletal (Msk), Cardiovascular and Respiratory (Car), Vasculitis (Vas), Renal (Ren) and Haematological (Hae). Time point of flare is represented with 13 patients’ samples (patients C4, C5, C8, C10, C13, C15, C16, C17, C18, C19, C20, C22 and C24). Follow-up group consists of patients’ samples from time-point of 3 months (patients C4, C5, C13, C15, C16, C17, C18 and C24) and time-point of 12 months post treatment (patients C8, C10, C19, C20 and C22).
(XLS)

Table S2 **Statin administration in SLE patients.** Five SLE patients out of thirteen received cholesterol-lowering therapy.
(XLS)

## References

[1] Mok CC, Lau CS (2003) Pathogenesis of systemic lupus erythematosus. J Clin Pathol 56: 481–490. 12835292 10.1136/jcp.56.7.481 PMC1769989

[2] Tenbrock K, Juang YT, Kyttaris VC, Tsokos GC (2007) Altered signal transduction in SLE T cells. Rheumatology (Oxford) 46: 1525–1530. 17586862 10.1093/rheumatology/kem154

[3] Jenks SA, Sanz I (2009) Altered B cell receptor signaling in human systemic lupus erythematosus. Autoimmun Rev 8: 209–213. 18723129 10.1016/j.autrev.2008.07.047 PMC2693706

[4] Pascual V, Banchereau J, Palucka AK (2003) The central role of dendritic cells and interferon-alpha in SLE. Curr Opin Rheumatol 15: 548–556. 12960479 10.1097/00002281-200309000-00005

[5] Kirou KA, Salmon JE, Crow MK (2006) Soluble mediators as therapeutic targets in systemic lupus erythematosus: cytokines, immunoglobulin receptors, and the complement system. Rheum Dis Clin North Am 32: 103–119, ix. 10.1016/j.rdc.2005.12.001 16504824

[6] Munoz LE, Gaipl US, Herrmann M (2008) Predictive value of anti-dsDNA autoantibodies: importance of the assay. Autoimmun Rev 7: 594–597. 18603024 10.1016/j.autrev.2008.06.003

[7] Munoz LE, Lauber K, Schiller M, Manfredi AA, Herrmann M (2010) The role of defective clearance of apoptotic cells in systemic autoimmunity. Nat Rev Rheumatol 6: 280–289. 20431553 10.1038/nrrheum.2010.46

[8] Gaipl US, Kuhn A, Sheriff A, Munoz LE, Franz S, et al (2006) Clearance of apoptotic cells in human SLE. Curr Dir Autoimmun 9: 173–187. 16394661 10.1159/000090781

[9] Niederer HA, Willcocks LC, Rayner TF, Yang W, Lau YL, et al (2010) Copy number, linkage disequilibrium and disease association in the FCGR locus. Hum Mol Genet 19: 3282–3294. 20508037 10.1093/hmg/ddq216 PMC2908468

[10] Bjorkhem I, Meaney S, Diczfalusy U (2002) Oxysterols in human circulation: which role do they have? Curr Opin Lipidol 13: 247–253. 12045393 10.1097/00041433-200206000-00003

[11] Brown AJ, Jessup W (2009) Oxysterols: Sources, cellular storage and metabolism, and new insights into their roles in cholesterol homeostasis. Mol Aspects Med 30: 111–122. 19248801 10.1016/j.mam.2009.02.005

[12] Lutjohann D, Breuer O, Ahlborg G, Nennesmo I, Siden A, et al (1996) Cholesterol homeostasis in human brain: evidence for an age-dependent flux of 24S-hydroxycholesterol from the brain into the circulation. Proc Natl Acad Sci U S A 93: 9799–9804. 8790411 10.1073/pnas.93.18.9799 PMC38509

[13] Bjorkhem I, Lutjohann D, Diczfalusy U, Stahle L, Ahlborg G, et al (1998) Cholesterol homeostasis in human brain: turnover of 24S-hydroxycholesterol and evidence for a cerebral origin of most of this oxysterol in the circulation. J Lipid Res 39: 1594–1600. 9717719

[14] Lutjohann D, Papassotiropoulos A, Bjorkhem I, Locatelli S, Bagli M, et al (2000) Plasma 24S-hydroxycholesterol (cerebrosterol) is increased in Alzheimer and vascular demented patients. J Lipid Res 41: 195–198. 10681402

[15] Leoni V, Masterman T, Diczfalusy U, De Luca G, Hillert J, et al (2002) Changes in human plasma levels of the brain specific oxysterol 24S-hydroxycholesterol during progression of multiple sclerosis. Neurosci Lett 331: 163–166. 12383922 10.1016/s0304-3940(02)00887-x

[16] Qin J, Goswami R, Balabanov R, Dawson G (2007) Oxidized phosphatidylcholine is a marker for neuroinflammation in multiple sclerosis brain. J Neurosci Res 85: 977–984. 17304573 10.1002/jnr.21206

[17] Seet RC, Lee CY, Lim EC, Tan JJ, Quek AM, et al (2010) Oxidative damage in Parkinson disease: Measurement using accurate biomarkers. Free Radic Biol Med 48: 560–566. 19969070 10.1016/j.freeradbiomed.2009.11.026

[18] Berthier A, Lemaire-Ewing S, Prunet C, Montange T, Vejux A, et al (2005) 7-Ketocholesterol-induced apoptosis. Involvement of several pro-apoptotic but also anti-apoptotic calcium-dependent transduction pathways. FEBS J 272: 3093–3104. 15955068 10.1111/j.1742-4658.2005.04723.x

[19] Kritharides L, Kus M, Brown AJ, Jessup W, Dean RT (1996) Hydroxypropyl-beta-cyclodextrin-mediated efflux of 7-ketocholesterol from macrophage foam cells. J Biol Chem 271: 27450–27455. 8910326 10.1074/jbc.271.44.27450

[20] Li W, Johnson H, Yuan XM, Jonasson L (2009) 7beta-hydroxycholesterol induces natural killer cell death via oxidative lysosomal destabilization. Free Radic Res 43: 1072–1079. 19707922 10.1080/10715760903176919

[21] McKinney EF, Lyons PA, Carr EJ, Hollis JL, Jayne DR, et al.. (2010) A CD8+ T cell transcription signature predicts prognosis in autoimmune disease. Nat Med 16: 586–591, 581p following 591. 10.1038/nm.2130 PMC3504359 20400961

[22] Isenberg DA, Rahman A, Allen E, Farewell V, Akil M, et al (2005) BILAG 2004. Development and initial validation of an updated version of the British Isles Lupus Assessment Group’s disease activity index for patients with systemic lupus erythematosus. Rheumatology (Oxford) 44: 902–906. 15814577 10.1093/rheumatology/keh624

[23] Folch J, Lees M, Sloane Stanley GH (1957) A simple method for the isolation and purification of total lipides from animal tissues. J Biol Chem 226: 497–509. 13428781

[24] Kim JH, Jittiwat J, Ong WY, Farooqui AA, Jenner AM (2010) Changes in cholesterol biosynthetic and transport pathways after excitotoxicity. J Neurochem 112: 34–41. 19860851 10.1111/j.1471-4159.2009.06449.x

[25] Cheng D, Jenner AM, Shui G, Cheong WF, Mitchell TW, et al (2011) Lipid pathway alterations in Parkinson’s disease primary visual cortex. PLoS One 6: e17299. 21387008 10.1371/journal.pone.0017299 PMC3046155

[26] Bligh EG, Dyer WJ (1959) A rapid method of total lipid extraction and purification. Can J Biochem Physiol 37: 911–917. 13671378 10.1139/o59-099

[27] Shui G, Stebbins JW, Lam BD, Cheong WF, Lam SM, et al (2011) Comparative plasma lipidome between human and cynomolgus monkey: are plasma polar lipids good biomarkers for diabetic monkeys? PLoS One 6: e19731. 21573191 10.1371/journal.pone.0019731 PMC3087804

[28] Tan EM, Cohen AS, Fries JF, Masi AT, McShane DJ, et al (1982) The 1982 revised criteria for the classification of systemic lupus erythematosus. Arthritis Rheum 25: 1271–1277. 7138600 10.1002/art.1780251101

[29] Hay EM, Bacon PA, Gordon C, Isenberg DA, Maddison P, et al (1993) The BILAG index: a reliable and valid instrument for measuring clinical disease activity in systemic lupus erythematosus. Q J Med 86: 447–458. 8210301

[30] Isenberg DA, Gordon C (2000) From BILAG to BLIPS–disease activity assessment in lupus past, present and future. Lupus 9: 651–654. 11199918 10.1191/096120300672904669

[31] Bombardier C, Gladman DD, Urowitz MB, Caron D, Chang CH (1992) Derivation of the SLEDAI. A disease activity index for lupus patients. The Committee on Prognosis Studies in SLE. Arthritis Rheum 35: 630–640. 1599520 10.1002/art.1780350606

[32] Alarcon GS, Roseman J, Bartolucci AA, Friedman AW, Moulds JM, et al (1998) Systemic lupus erythematosus in three ethnic groups: II. Features predictive of disease activity early in its course. LUMINA Study Group. Lupus in minority populations, nature versus nurture. Arthritis Rheum 41: 1173–1180. 9663472 10.1002/1529-0131(199807)41:7<1173::AID-ART5>3.0.CO;2-A

[33] Smith MF Jr, Hiepe F, Dorner T, Burmester G (2009) Biomarkers as tools for improved diagnostic and therapeutic monitoring in systemic lupus erythematosis. Arthritis Res Ther 11: 255. 19939293 10.1186/ar2834 PMC3003542

[34] Francis L, Perl A (2009) Pharmacotherapy of systemic lupus erythematosus. Expert Opin Pharmacother 10: 1481–1494. 19505215 10.1517/14656560902971003

[35] Liu CC, Manzi S, Ahearn JM (2005) Biomarkers for systemic lupus erythematosus: a review and perspective. Curr Opin Rheumatol 17: 543–549. 16093831 10.1097/01.bor.0000174182.70159.22

[36] Smith RM, Clatworthy MR, Jayne DR (2010) Biological therapy for lupus nephritis-tribulations and trials. Nat Rev Rheumatol 6: 547–552. 20647994 10.1038/nrrheum.2010.117

[37] Bjorkhem I, Lovgren-Sandblom A, Piehl F, Khademi M, Pettersson H, et al (2011) High levels of 15-oxygenated steroids in circulation of patients with multiple sclerosis: fact or fiction? J Lipid Res 52: 170–174. 20934989 10.1194/jlr.D011072 PMC2999927

[38] Griffiths WJ, Wang Y (2010) Are 15-oxygenated sterols present in the human circulation? J Lipid Res 52: 4–5. 20956549 10.1194/jlr.E012088 PMC2999921

[39] Bjorkhem I, Diczfalusy U, Olsson T, Russell DW, McDonald JG, et al.. (2011) Detecting oxysterols in the human circulation. Nat Immunol 12: 577; author reply 577–578. 10.1038/ni0711-577a 21685947

[40] Descloux E, Durieu I, Cochat P, Vital Durand D, Ninet J, et al (2008) Paediatric systemic lupus erythematosus: prognostic impact of antiphospholipid antibodies. Rheumatology (Oxford) 47: 183–187. 18160418 10.1093/rheumatology/kem335

[41] Sorice M, Circella A, Misasi R, Pittoni V, Garofalo T, et al (2000) Cardiolipin on the surface of apoptotic cells as a possible trigger for antiphospholipids antibodies. Clin Exp Immunol 122: 277–284. 11091286 10.1046/j.1365-2249.2000.01353.x PMC1905767

[42] Morrow JD, Hill KE, Burk RF, Nammour TM, Badr KF, et al (1990) A series of prostaglandin F2-like compounds are produced in vivo in humans by a non-cyclooxygenase, free radical-catalyzed mechanism. Proc Natl Acad Sci U S A 87: 9383–9387. 2123555 10.1073/pnas.87.23.9383 PMC55169

[43] Basu S (2008) F2-isoprostanes in human health and diseases: from molecular mechanisms to clinical implications. Antioxid Redox Signal 10: 1405–1434. 18522490 10.1089/ars.2007.1956

[44] Smith PP, Gordon C (2010) Systemic lupus erythematosus: clinical presentations. Autoimmun Rev 10: 43–45. 20850569 10.1016/j.autrev.2010.08.016

[45] Bilzer M, Roggel F, Gerbes AL (2006) Role of Kupffer cells in host defense and liver disease. Liver Int 26: 1175–1186. 17105582 10.1111/j.1478-3231.2006.01342.x

[46] Kosugi I, Muro H, Shirasawa H, Ito I (1992) Endocytosis of soluble IgG immune complex and its transport to lysosomes in hepatic sinusoidal endothelial cells. J Hepatol 16: 106–114. 1484143 10.1016/s0168-8278(05)80102-3

[47] Gronwall C, Akhter E, Oh C, Burlingame RW, Petri M, et al (2012) IgM autoantibodies to distinct apoptosis-associated antigens correlate with protection from cardiovascular events and renal disease in patients with SLE. Clin Immunol 142: 390–398. 22297166 10.1016/j.clim.2012.01.002 PMC3632049

[48] Mehrani T, Petri M (2011) IgM anti-beta2 glycoprotein I is protective against lupus nephritis and renal damage in systemic lupus erythematosus. J Rheumatol 38: 450–453. 21123325 10.3899/jrheum.100650

[49] Leoni V, Masterman T, Mousavi FS, Wretlind B, Wahlund LO, et al (2004) Diagnostic use of cerebral and extracerebral oxysterols. Clin Chem Lab Med 42: 186–191. 15061359 10.1515/CCLM.2004.034

[50] Shafaati M, Marutle A, Pettersson H, Lovgren-Sandblom A, Olin M, et al.. (2011) Marked accumulation of 27-hydroxycholesterol in the brain of Alzheimer patients with the Swedish APP 670/671 mutation. J Lipid Res. 10.1194/jlr.M014548 PMC3073459 21335619

[51] Schule R, Siddique T, Deng HX, Yang Y, Donkervoort S, et al (2010) Marked accumulation of 27-hydroxycholesterol in SPG5 patients with hereditary spastic paresis. J Lipid Res 51: 819–823. 19812052 10.1194/jlr.M002543 PMC2842155

[52] Yasunobu Y, Hayashi K, Shingu T, Yamagata T, Kajiyama G, et al (2001) Coronary atherosclerosis and oxidative stress as reflected by autoantibodies against oxidized low-density lipoprotein and oxysterols. Atherosclerosis 155: 445–453. 11254916 10.1016/s0021-9150(00)00581-5

[53] Leoni V, Lutjohann D, Masterman T (2005) Levels of 7-oxocholesterol in cerebrospinal fluid are more than one thousand times lower than reported in multiple sclerosis. J Lipid Res 46: 191–195. 15576852 10.1194/jlr.C400005-JLR200

[54] Navarra SV, Ishimori ML, Uy EA, Hamijoyo L, Sama J, et al.. (2010) Studies of Filipino patients with systemic lupus erythematosus (SLE): Autoantibody profile of first degree relatives. Lupus. 10.1177/0961203310385164 21183559

[55] Fusaro AE, Fahl K, Cardoso EC, de Brito CA, Jacob CM, et al (2010) Profile of autoantibodies against phosphorylcholine and cross-reactivity to oxidation-specific neoantigens in selective IgA deficiency with or without autoimmune diseases. J Clin Immunol 30: 872–880. 20737202 10.1007/s10875-010-9453-y

[56] Ortona E, Capozzi A, Colasanti T, Conti F, Alessandri C, et al (2010) Vimentin/cardiolipin complex as a new antigenic target of the antiphospholipid syndrome. Blood 116: 2960–2967. 20634382 10.1182/blood-2010-04-279208

[57] Abou-Raya A, el-Hallous D, Fayed H (2004) 8-Isoprostaglandin F2 alpha: a potential index of lipid peroxidation in systemic lupus erythematosus. Clin Invest Med 27: 306–311. 15675110

[58] Ames PR, Alves J, Murat I, Isenberg DA, Nourooz-Zadeh J (1999) Oxidative stress in systemic lupus erythematosus and allied conditions with vascular involvement. Rheumatology (Oxford) 38: 529–534. 10402073 10.1093/rheumatology/38.6.529

[59] Szodoray P, Tarr T, Tumpek J, Kappelmayer J, Lakos G, et al (2009) Identification of rare anti-phospholipid/protein co-factor autoantibodies in patients with systemic lupus erythematosus. Autoimmunity 42: 497–506. 19626489 10.1080/08916930902882731

[60] Zuliani G, Donnorso MP, Bosi C, Passaro A, Dalla Nora E, et al (2011) Plasma 24S-hydroxycholesterol levels in elderly subjects with late onset Alzheimer’s disease or vascular dementia: a case-control study. BMC Neurol 11: 121. 21970714 10.1186/1471-2377-11-121 PMC3199239

