# Correction: Lipid Anti-Lipid Antibody Responses Correlate with Disease Activity in Systemic Lupus Erythematosus

**DOI:** 10.1371/annotation/73d18051-4c35-4c45-ad26-7c670de76db0

**Published:** 2013-10-16

**Authors:** Vojislav Jovanović, Nurhuda Abdul Aziz, Yan Ting Lim, Amanda Ng Ai Poh, Sherlynn Jin Hui Chan, Eliza Ho Xin Pei, Fei Chuin Lew, Guanghou Shui, Andrew M. Jenner, Li Bowen, Eoin F. McKinney, Paul A. Lyons, David M. Kemeny, Kenneth G. C. Smith, Markus R. Wenk, Paul A. MacAry

The thirteenth author's name was spelled incorrectly. The correct name is David M. Kemeny.

